# Molecular and Cellular Response Profiles Induced by the TLR4 Agonist-Based Adjuvant Glucopyranosyl Lipid A

**DOI:** 10.1371/journal.pone.0051618

**Published:** 2012-12-28

**Authors:** Stacie L. Lambert, Chin-Fen Yang, Zheng Liu, Rosemary Sweetwood, Jackie Zhao, Lily Cheng, Hong Jin, Jennifer Woo

**Affiliations:** MedImmune LLC, Mountain View, California, United States of America; St.Louis University, United States of America

## Abstract

**Background:**

Toll-like receptor (TLR)4 agonists are known potent immunostimulatory compounds. These compounds can be formulated as part of novel adjuvants to enhance vaccine medicated immune responses. However, the contribution of the formulation to the innate *in vivo* activity of TLR4 agonist compounds is not well understood.

**Methodology and Principal Findings:**

We evaluated synthetic TLR4 agonist Glucopyranosyl Lipid A (GLA) for its effects on molecular and cellular innate immune responses in the murine model. Microarray techniques were used to compare the responses to GLA in an aqueous formulation or in an oil-in-water Stable Emulsion formulation (GLA-SE) versus either SE alone or the mineral salt aluminum hydroxide (alum) at the muscle injection site over multiple timepoints. In contrast to the minimal gene upregulation induced by SE and alum, both GLA and GLA-SE triggered MyD88- and TRIF-dependent gene expression. Genes for chemokines, cytokine receptors, signaling molecules, complement, and antigen presentation were also strongly upregulated by GLA and GLA-SE. These included chemokines for T_H_1-type T cells (CXCL9 and CXCL10) and mononuclear leukocytes (CCL2, CCL3) among others. GLA-SE induced stronger and more sustained gene upregulation than GLA in the muscle; GLA-SE induced genes were also detected in local draining lymph nodes and at lower levels in peripheral blood. Both GLA and GLA-SE resulted in increased cellular trafficking to the draining lymph nodes and upregulated MHC molecules and ICAM1 on local dendritic cells. GLA and GLA-SE transiently upregulated circulating MCP-1, TNFα, IFNγ and IP-10 in blood.

**Conclusions/Significance:**

While GLA and GLA-SE activate a large number of shared innate genes and proteins, GLA-SE induces a quantitatively and qualitatively stronger response than GLA, SE or alum. The genes and proteins upregulated could be used to facilitate selection of appropriate adjuvant doses in vaccine formulations.

## Introduction

A diverse set of compounds can act as vaccine adjuvants to enhance immune responses to coadministered protein antigens. Mineral salts of aluminum hydroxide and aluminum phosphate (both known as alum) have been used in a large number of approved human vaccines. Squalene oil-in-water emulsions such as MF59 and AS03 are approved adjuvants for human influenza vaccines in Europe. Another squalene-oil emulsion known as Stable Emulsion (SE) has been shown to enhance antibody responses in human subjects [1]. Both alum and squalene-oil based adjuvants tend to bias towards T helper (T_H_) 2 rather than T_H_1 immunity [1]. T_H_1 immunity and associated cellular immune responses are important host defense mechanism against intracellular pathogens. Pathogen-derived components such as lipopolysaccharide (LPS) and its mimics have robust innate immunostimulatory effects through Toll like receptor (TLR)4-dependent mechanisms that can promote T_H_1 responses [2]. AS04 (an alum-adsorbed purified detoxified monophosphoryl lipid A, MPL) is the first of a new generation of adjuvants incorporating TLR4 agonists and has been approved for use in human papillomavirus (HPV) and Hepatitis B vaccines [3]. MPL in combination with alum induces T_H_1 bias response in mouse models [4]. A related TLR4 agonist, the synthetic hexaacylated lipid A derivative Glucopyranosyl Lipid A (GLA) formulated with SE, also induces T_H_1 biased responses and has been evaluated as an adjuvant in influenza vaccine clinical trials [5].

Recent technology advances have made it possible to understand the molecular mechanisms responsible for the activity of commercially approved adjuvants. For example, alum's adjuvant activity is no longer considered to be solely through a “depot” effect in retaining antigen at the site of injection. In fact, alum induces cell death and endogenous danger signals that trigger inflammation pathways and activates the NALP3 inflammasome pathway to generate active IL-1β and IL-18 from precursors (reviewed in [6]). Recent transcriptional profiling studies in mouse muscle indicated that alum weakly induced cytokine genes such as CCL2, CCL6, CCL7 and CXCL10 as well as major histocompatability complex (MHC) genes [7]. In the same study, MF59 was found to induce transcription of cytokine genes such as CCL2, CCL6, CCL7, CCL9, CXCL5, and CXCL10. Transcriptional profiles of other oil-in-water emulsions have not been reported, but AS03 was found to induce production of the cytokine proteins IL-6, CXCL1, and CSF3 [8]. These cytokines promote recruitment of antigen presenting cells to the site of vaccination and to draining lymph nodes [7], [8].

To our knowledge, the *in vivo* innate transcription profiles of TLR4 agonists, whether given in aqueous form or formulated with an emulsion, have not been described. It has recently been demonstrated *in vitro* that GLA and MPL induce gene transcription of cytokines such as IL-6, TNFα, CXCL1, and CXCL10 in both mouse and human dendritic cells [9]. A separate study reported increased IL-6, TNFα, CCL2 and CCL3 in injected mouse muscle in response to MPL and MPL + alum compared to alum but a transcriptional evaluation was not included in that study [4].

In this study, we characterized the kinetics of gene transcription and serum cytokine expression following intramuscular immunization of mice with GLA or GLA-SE in comparison to SE or alum. GLA-SE induced the strongest responses both in levels and in duration of gene transcription with a similar profile between muscle and draining lymph nodes. Although fewer genes were activated in the peripheral blood, GLA-SE resulted in higher levels of serum cytokines. In addition, GLA-SE induced maximal recruitment and activation of dendritic cells in draining lymph nodes. Overall, the GLA-SE formulation induces a qualitatively and quantitatively stronger innate immune response than GLA, SE or alum.

## Results

### GLA-SE stimulated robust and sustained gene transcription at the site of injection

**Figure 1:**
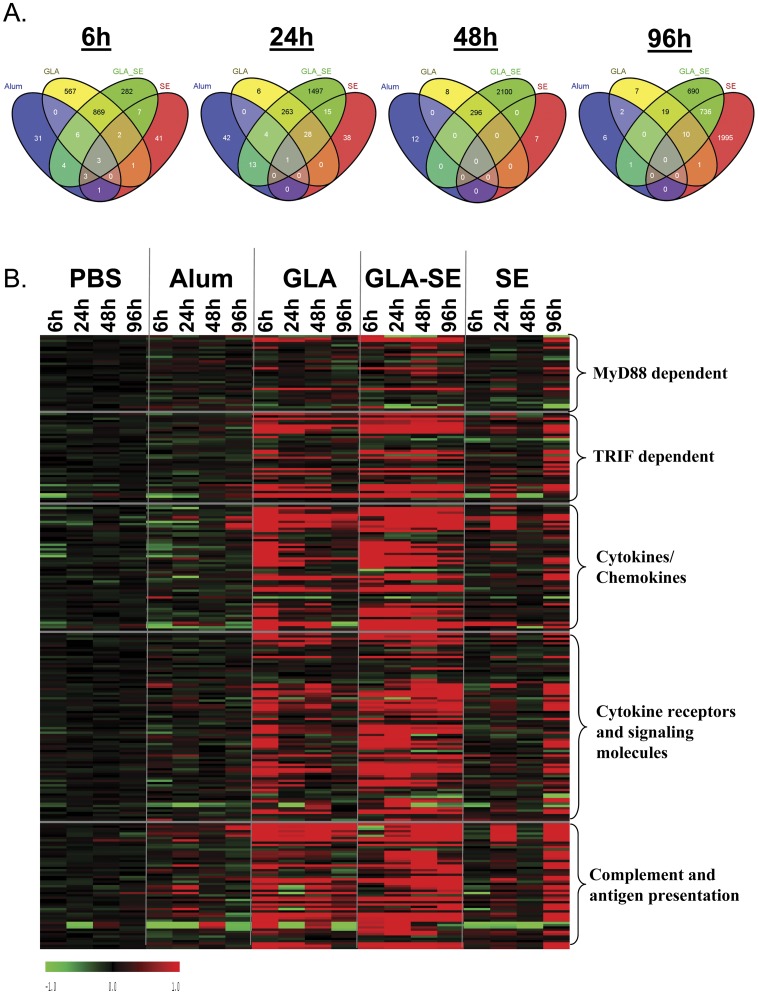
Gene expression profile in adjuvant treated groups. (A) Venn diagram showed differentially over-expressed genes in the muscle following intramuscular inoculation with alum, GLA, GLA-SE or SE at each time point. A cut-off of 2-fold or greater expression than PBS control was used to identify over-expressed genes. (B) Heatmap showing expression profile of selected genes in muscle following intramuscular inoculation with PBS, alum, GLA, GLA-SE or SE at each time point. Red indicates higher expression, while green indicates lower expression compared to baseline. Data shown is the mean of n = 3 individual animal samples per timepoint and treatment group, each run in duplicate, from one of two experiments.

Induction of gene expression at the muscle site of injection was compared over a time course for GLA, GLA-SE, SE, and alum adjuvants. The number of GLA induced transcripts peaked at 6 hours with 880 genes shared with the GLA-SE treatment group (61% of the total genes induced by GLA and 74% of those induced by GLA-SE) (Figure 1A). The peak time point of GLA-SE induced gene expression in the muscle was at 48 hours post inoculation, with a total of 2396 upregulated gene transcripts. While greater than 97% of the genes induced by GLA at 24 and 48 hours were shared with GLA-SE, less than 20% of the genes induced by GLA-SE at 24 and 48 hours were shared with GLA. Many of these were unique transcripts not induced by other treatment groups. SE induced gene transcription at a later time, with a peak number of genes observed at 96 hours. At the 96 hour timepoint, over 50% of the genes induced by GLA-SE were also induced by SE, indicating transcriptional regulation by the emulsion component, SE. In contrast to the other adjuvants tested, alum induced a very small number of gene transcripts (maximum 60 at 24 hours), with the majority not induced by other tested adjuvants.

Consistent with our understanding of TLR4 signaling through MyD88/MAL pathways to induce NFκB-dependent genes and through TRIF/TRAM pathways to induce interferon family genes [10], the TLR4 agonists GLA and GLA-SE induced transcriptional upregulation of both MyD88 and TRIF dependent genes *in vivo* as early as 6 hours post injection (Figure 1B). TRIF-dependent genes were upregulated to a greater extent than MyD88 dependent genes. The most strongly induced transcripts included cytokines/chemokines, cytokine receptors and signaling molecules, and molecules involved in complement and antigen presentation pathways.

CXCL9 and CXCL10 were the two most highly upregulated cytokine mRNAs in the muscle of mice treated with GLA or GLA-SE. CXCL9 and CXCL10 play key roles in the recruitment of activated T_H_1-type T cells [11], and the high sustained levels of these cytokines may predispose a T_H_1-type immune response to GLA or GLA-SE adjuvanted antigens. In the GLA treatment group, CXCL9 and CXCL10 transcripts peaked at 6 hours at levels of 116- and 227-fold higher than the PBS treated animals, respectively (Table S1). CXCL9 levels dropped to 33-fold at 24 hours, then to 4-fold at 96 hour. CXCL10 levels dropped to 9-fold by 24 hours and declined to baseline levels by 48 hours (Table S1). In the GLA-SE treatment group, CXCL9 was high at 6 hours (94-fold), peaked at 24 hours (311-fold) and slowly decreased to 6-fold by 96 hours. CXCL10 peaked at 6 hours (175-fold), stayed elevated at 24 and 48 hours (79- and 28-fold, respectively) and declined to baseline levels by 96 hours. In comparison, a B cell recruiting chemokine, CXCL13, was persistently upregulated over the entire 96 hr time course in both GLA (21-fold at 24 hours) and GLA-SE (44-fold at 24 hours) treatment groups.

In addition to inducing T and B cell chemokine expression, GLA and GLA-SE were found to rapidly (peaking at the 6 hour timepoint) induce high levels of chemokine genes involved in recruitment and activation of antigen presenting cells (APC). These included the neutrophil-recruiting chemokines CXCL1 (82- and 235-fold induced, respectively), CXCL2 (58- and 116-fold induced, respectively), and CXCL5 (23- and 95-fold induced, respectively) [11]. Other upregulated cytokines were monocyte-recruiting chemokines CCL2 (55- and 56-fold induced, respectively) and CCL3 (19- and 45-fold induced, respectively). Macrophage-related CCL6 and CCL9 chemokines were also induced by both treatments, but their levels and kinetics were different between these two treatment groups. CCL6 and CCL9 peaked at 6 hours (both at ∼8-fold baseline) in the GLA treatment group, but reached much higher peak levels at 96 hours (49- and 35-fold baseline) in the GLA-SE treatment group. The pro-inflammatory cytokine IL-6 which is involved in APC activation was also upregulated (peaking at 6 hours at 64- and 169- fold induced, respectively). However, inflammatory cytokines such as TNFα were not significantly induced by either of these treatments.

In contrast to GLA or GLA-SE, alum and SE induced weak cytokine gene responses. CCL9 was the only cytokine gene induced by alum with a level induced by only 2-fold (Table S1). SE-induced CXCL9 and CXCL13 transcripts peaked at 96 hours (27- and 11-fold, respectively). CCL6 and CCL9 transcripts were also induced by SE, peaking at 24 hours (9.2- and 7.4- fold, respectively).

Chemokine receptors found on APC or T cells were also highly induced in muscle by GLA and GLA-SE, with GLA-SE being more potent. CCR1, CCR2, and CSF1R are found on monocytes and other antigen presenting cells, while CCR5 is found on T_H_1 T cells [11]. The peak levels of CCR1, CCR2, CCR5 and CSF1R were 9.5-, 10.7-, 8.8- and 3.4-fold with GLA treatment, compared to 14.1-, 26.2-, 28.7- and 15.8-fold by GLA-SE treatment, respectively (Table S1). Alum did not upregulate these transcripts. SE induced these transcripts at moderate levels: CCR1 (2.0-fold), CCR2 (3.8-fold), CCR5 (2.9-fold), and CSF1R (9.8-fold).

Genes involved in antigen presentation by APC to T cells were upregulated in muscle in both the GLA and GLA-SE treatment groups. MHC I (H2-D1 and H2-K1) and MHC II (H2-AA and H2-EA) gene transcripts were 3–7-fold upregulated at 6 hours post GLA treatment but dropped to baseline levels by 24 hours (Table S1). Following GLA-SE administration, MHC I gene transcripts were 4–5-fold upregulated at 6 hours with a peak level of 10-11-fold upregulated at 48 hours. MHC II gene transcripts were only detected at 96 hours post GLA-SE treatment (12–15-fold upregulated). In contrast, alum had no effect on MHC I gene expression and induced MHC II gene expression weakly (∼3-fold at 24 hours). SE treatment had little effect on either MHC I or MHC II gene transcription at early timepoints (6–48 hours) but strongly induced these molecules (10–12-fold) at 96 hours. These different MHC expression patterns may affect the subtypes of T cells activated in response to antigens formulated with each adjuvant, with more CD4 T cells activated by MHC II-enhancing adjuvants and more CD8 T cells activated by MHC I-enhancing adjuvants.

### Enhanced recruitment of immune cells to local muscle tissues by GLA-SE

**Figure 2:**
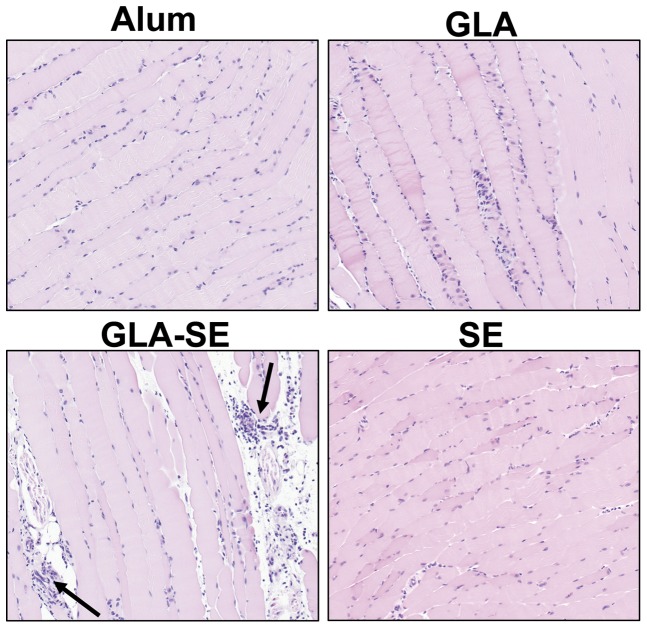
Histological evaluation of muscle injection site. Representative H&E stained muscle sections (10x) from alum, GLA, GLA-SE or SE treated animals at the 24 hour time point. The arrows in the GLA-SE group indicate areas of immune cell infiltrate into the muscle fascia.

Myocytes are known to express TLR4 and may be directly activated by GLA-containing adjuvants [12]. Enhanced transcription levels of individual genes in the muscle injection site could be due to gene expression by local tissues or by chemokine-recruited leukocytes. Histopathologic evaluation of leukocytic infiltration in the muscle was thus compared for each treatment group at each time point. Mild cellular infiltration by neutrophils admixed with fewer lymphocytes was seen in the perimuscular connective tissue fascia (indicated by arrows) in the GLA-SE treatment group at each timepoint (Figure 2). A single animal in the GLA treatment group showed even milder cellular infiltration at 6 hours. No significant cellular infiltrate was observed in SE or alum treatment groups at any timepoint. While recruited activated leukocytes may be responsible for some of the enhanced gene transcription observed in the GLA-SE treatment group, local myocytes directly activated by GLA, SE, or alum adjuvants likely contribute to enhanced transcription in those groups.

### GLA-SE stimulated distinct gene transcription profiles in draining lymph nodes and blood

**Figure 3:**
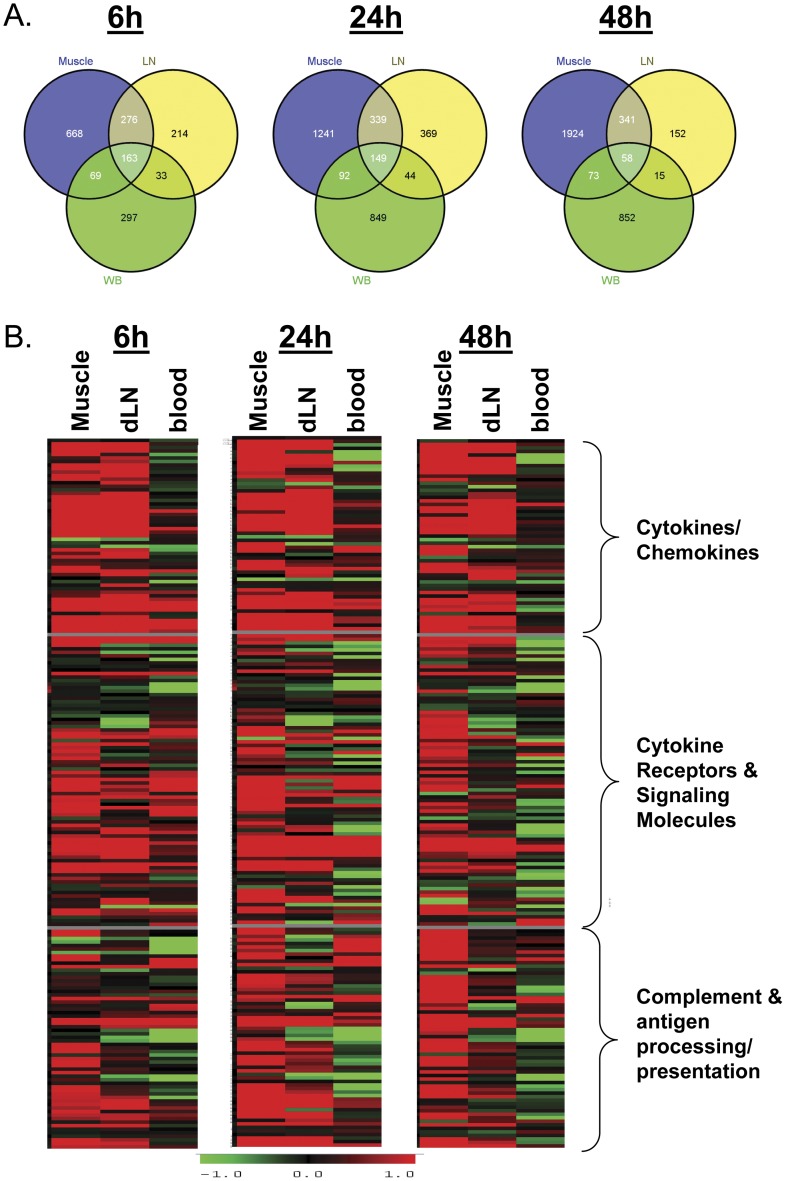
Comparison of GLA-SE treated group for gene expression in muscle, dLN and blood. (A) Venn diagram showing differentially over-expressed genes among muscle, draining lymph node (LN), and blood (WB) harvested at 6, 24, or 48 hours following intramuscular inoculation with GLA-SE. (B) Heat maps showing expression profile of selected genes in muscle, draining lymph node (dLN) and blood at 6, 24, or 48 hours following intramuscular inoculation with GLA-SE. Red: higher expression; green: lower expression compared to baseline (PBS control values at each timepoint). Data shown is the mean of n = 3 individual animal samples per timepoint and treatment group, each run in duplicate. Muscle data is from the same experiment as shown in Figure 1.

Adjuvants may have transcriptional effects at sites remote from the site of injection, such as in draining lymph nodes (dLN) or peripheral blood. In this study, induction of gene expression at these sites was compared with that seen in muscle at different timepoints following GLA-SE treatment. Overall, more total gene transcripts were upregulated in muscle (1176–2396) than in dLN (566–901) or blood (562–1134) in response to GLA-SE (Figure 3A). The majority of individual gene transcripts upregulated in muscle were not upregulated in either dLN or blood. The gene transcripts upregulated in dLN were primarily ones that were also upregulated in muscle (54–70% shared). In contrast, the gene transcripts upregulated in blood were primarily unique transcripts that were not observed in muscle or dLN. Individual genes shared by all three tissues ranged from a high of 163 at 6 hours to a low of 58 at 48 hours.

Both injected compounds and activated leukocytes can rapidly traffic from peripheral muscles to dLN, which might account for the large percentage of upregulated transcripts in dLN shared with muscle. Thus, the transcriptional levels of selected genes among muscle, dLN, and blood were compared. The highest transcription levels following GLA-SE treatment were observed in muscle, with less in dLN and minimal levels in blood (Figure 3B). Upregulation of cytokine gene transcripts associated with T cell and APC recruitment in muscle and dLN were often quite similar, with CCL2, CSF3, CXCL2, CXCL10, IL-1β and IL-6 levels in muscle and dLN within 2-fold of each other at 6 hours post GLA-SE treatment. However, CCL5, CXCL1, CXCL5 and CXCL9 had more than 2-fold higher induction in muscle compared to dLN; and CCL3, CCL4, CXCL11, and IFNγ had more than 2-fold higher induction in dLN compared to muscle (Table S2). Cell surface receptor gene transcripts such as CCR2, CCR5, IL-6RA, IL-13RA1, B2M, MHC I and MHC II transcripts were often lower in dLN than muscle. In contrast, CCR1 was similarly induced in muscle and dLN, while FcγR1 was 2-fold higher in dLN compared to muscle at early timepoints.

In general, blood had minimal transcriptional upregulation. Only a small number of cytokine genes such as CXCL2, CXCL10, and TNFαwere found weakly upregulated at 6 hours in blood (Figure 3B). The two cytokines that were more strongly upregulated in blood than in other tissues were Spp1 (osteopontin), which acts on monocytes to enhance T_H_1-type responses [13] and was upregulated at the 6 hour timepoint; and CXCL13, which was upregulated at the 24 hour timepoint (Table S2). Transcripts for cytokine receptors CCR1, CCR5, and IL-15RA were weakly induced in blood at levels similar to muscle and dLN at the early 6 hour timepoint, while IL-13RA1 was induced in blood at levels similar to muscle but higher than dLN. IL-1R2 was one of the few cytokine receptor transcripts induced at higher levels in blood than muscle or dLN at early timepoints.

### GLA-SE induced enhanced dendritic cell recruitment to draining lymph nodes

**Figure 4:**
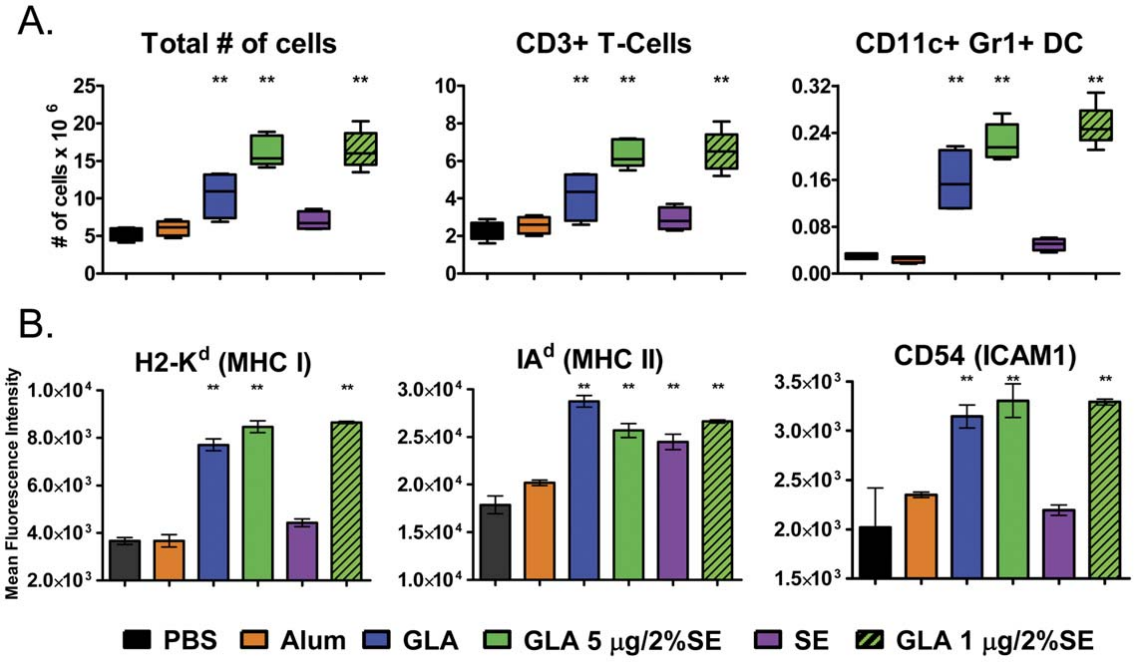
Cell recruitment to draining lymph nodes. (A) Numbers of cells recovered at 24 hours from dLN (iliac and popliteal) of mice treated with the indicated adjuvants. Left panels: total recovered cell counts; middle panels: numbers of CD3+ T cells; right panels: CD11c+ Gr1+ dendritic cells. Subset cell numbers were calculated by multiplying the percentage of specific cells gated by flow cytometric analysis by the total number of recovered cells. Data shown is the min-max and mean from n = 4–5 individual animals per group, evaluated in 2 replicate studies. ** indicates p<0.05 versus the placebo control group. (B) Mean fluorescence intensity (MFI) of surface expressed H2-K^d^ (MHC I) (left panel), IA^d^ (MHC II) (middle panel), and CD54 (ICAM1) (right panel) molecules on gated CD11c+Gr1+ dendritic cells recovered from draining lymph nodes at 24 hours following treatment of mice with the indicated adjuvants.

Transcriptional upregulation in dLN may be due to enhanced de novo gene expression or to an induced influx of specific immune cell types. We evaluated the number and types of immune cells present in dLN at 24 hours post injection with the previously tested PBS, alum, GLA, GLA-SE (5 μg GLA in 2% SE), and SE. In addition, a lower dose of GLA-SE (1 μg GLA in 2% SE) was also included. The 24 hour timepoint has been used by other researchers to evaluate immune recruitment [14]. We found that significantly more cells were recruited to dLN in groups that received GLA or GLA-SE compared to PBS. PBS-treated mice had ∼5×10^6^ dLN cells/mouse, while GLA treated mice had ∼10×10^6^ dLN cells/mouse and GLA-SE treated mice had ∼16×10^6^ dLN cells/mouse (Figure 4A). Interestingly, both doses of GLA-SE used (1 μg or 5 μg in 2% SE) had a similar effect on cell recruitment. Total CD3+ T cell numbers in dLN were increased from ∼2×10^6^ with PBS treatment to ∼4×10^6^ with GLA treatment and ∼6×10^6^ for either GLA-SE treatment group. In contrast, alum and SE had no significant impact on total dLN cell numbers or on T cell numbers. CD11c+ GR1+ dendritic cells (DC) in the lymph node are a small but important population that acts as antigen presenting cells for T cell activation. Their numbers were disproportionately increased in response to GLA or GLA-SE treatment, from ∼0.03×10^6^ with PBS to ∼0.15×10^6^ with GLA treatment and ∼0.22–.25×10^6^ with GLA-SE treatment. In contrast, alum and SE treatment had no significant impact on CD11c+ GR1+ DC numbers compared to PBS.

DC activation states can influence their ability to promote T and B cell responses. We evaluated mean fluorescence intensity (MFI) of antibody binding to key surface markers such as H2-K^d^ (MHC I), IA^d^ (MHC II), and ICAM1 on CD11c+ GR1+ DC as surrogate markers of DC activation (Figure 4B). DC H2-K^d^ MHC I levels increased more than 2-fold in the GLA and GLA-SE treatment groups (to 7.7×10^3^ MFI with GLA and 8.5–8.6×10^3^ MFI with GLA-SE). This correlates with the ∼2-fold induction of MHC I mRNA observed in dLN following GLA-SE treatment. DC IA^d^ MHC II levels were increased with GLA (1.6-fold), GLA-SE (1.4–1.5 fold), or SE treatment (1.3-fold), but to a lesser degree than MHC I. DC CD54 (ICAM1) levels were significantly increased following GLA (1.55-fold) or GLA-SE (1.65-fold) treatment. Both doses of GLA-SE used (1 μg or 5 μg in 2% SE) had a similar effect on DC expression of H2-K^d^, IA^d^, and ICAM.

### Cytokine responses to GLA-SE and other adjuvants were transiently detected in serum

**Figure 5:**
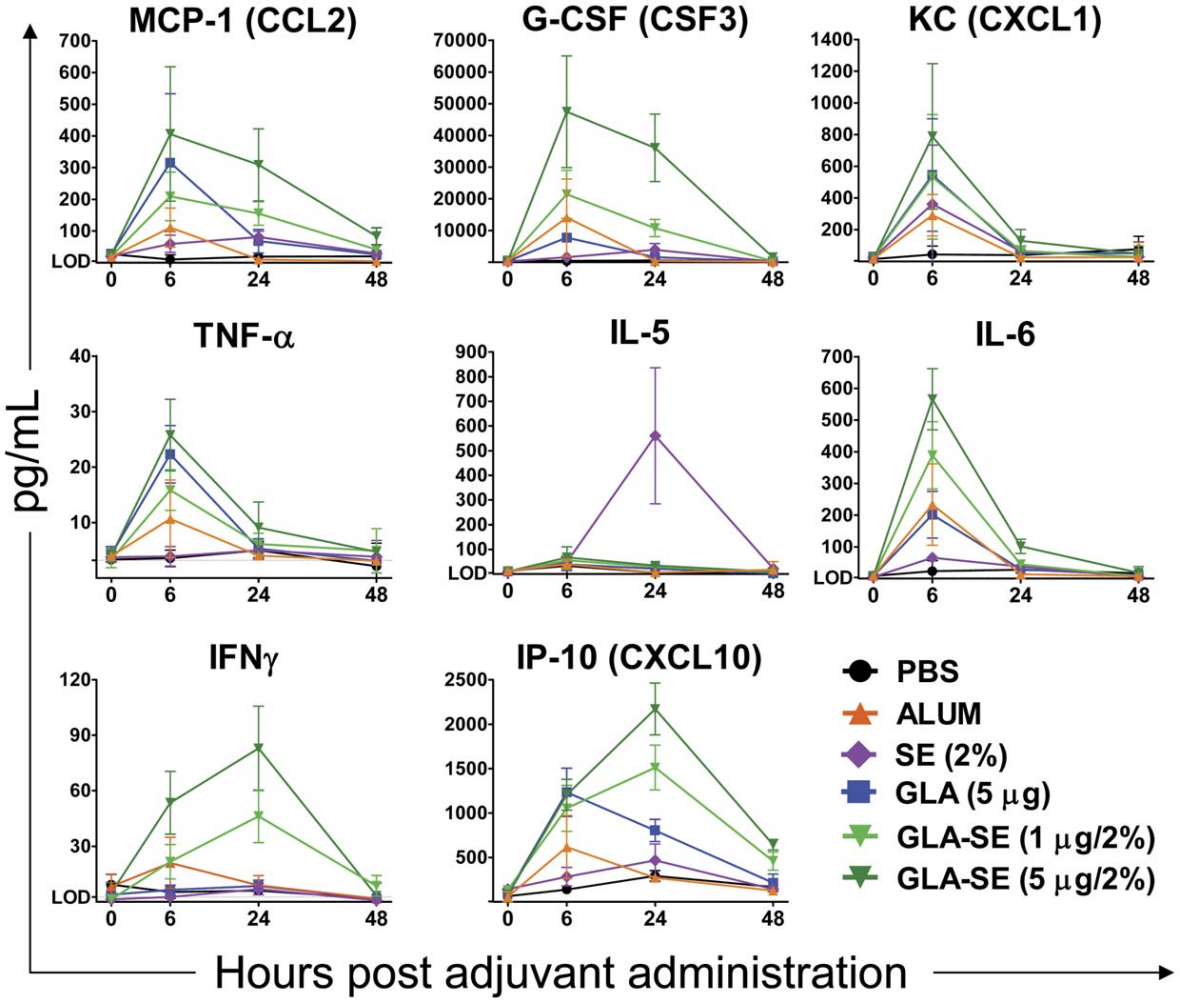
Serum cytokine induction. Levels of representative cytokines (CCL2, CSF3, CXCL1, TNFα, IL-5, IL-6, IFNγ, and CXCL10) were measured for each treatment group in the serum at 6–48 hours after adjuvant administration in 3–4 replicate experiments. Data is presented as the mean plus standard deviation of N = 8–12 for the 6 and 24 hour timepoints and N = 3 for the 48 hour timepoint.

Cytokines generated at the muscle injection site or the dLN can diffuse to other sites. Thus, adjuvant-induced cytokine proteins could be detected in the blood even if their mRNAs were not detected in the blood. We evaluated the kinetics of the serum cytokine responses in animals treated with each adjuvant and detected increased cytokine proteins in response to each adjuvant (Figure 5). Most evaluated cytokines peaked at 6 hours, including CCL2, CXCL1, TNFα, IL-6, and CSF3. However, T cell-related cytokines such as IFNγ and CXCL10 peaked at 24 hours. These responses were temporary, as all these cytokines returned to baseline in all groups by 48–96 hours.

Of the adjuvants examined in this study, GLA-SE induced the most robust serum cytokine response. By 6 hours following GLA-SE treatment, CCL2 (MCP-1), CCL4 (MIP-1β), CXCL1 (KC), CXCL9 (MIG), CSF3 (G-CSF), IFNγ IL-6 and IL-10 were upregulated more than 10-fold over the PBS group, with a number of other cytokines upregulated more than 2-fold (Table 1). GLA treatment induced the second most robust serum cytokine response, with particularly strong (>10-fold) induction of CCL2, CXCL1, CSF3, IL-6 and IL-10. In contrast, alum or SE induced at most an ∼8-fold increase in expression of any cytokine. Alum induced IL-6 moderately (8-fold), and CCL2, CCL3, CXCL1, IL-1α, IL-5, IL-13, IL-15, and TNFα weakly at ∼2-5-fold. SE also induced IL-6 moderately (8.1-fold) and CCL2, CCL3, CXCL1, CSF3, IL-5 and IL-13 at ∼2-5-fold. Among all the treatment groups, SE induced the highest IL-5 expression. This has also been seen with other emulsions such as MF59 [7].

**Table 1:** Effect of Adjuvant on Serum Cytokine and Chemokine Levels at 6 hours.

		*PBS*		*Alum*				*GLA*				*GLA-SE*				*SE*	
Cytokine/Chemokine		Median		Median		*Fold Change*		Median		*Fold Change*		Median		*Fold Change*		Median	*Fold Change*
CCL2 (MCP-1)	17.1		35.4		*2.1*		376.8		*22.0*		487.2		*28.4*		49.1		*2.9*
CCL3 (MIP-1α)	17.8		42.3		*2.4*		97.6		*5.5*		121.1		*6.8*		34.8		*2.0*
CCL4 (MIP-1β)	53.3		65.8		*1.2*		422.0		*7.9*		559.6		*10.5*		100.8		*1.9*
CCL5 (RANTES)	15.8		23.8		*1.5*		39.0		*2.5*		68.5		*4.3*		22.0		*1.4*
CXCL1 (KC)	37.9		94.9		*2.5*		431.0		*11.4*		1105.7		*29.2*		186.0		*4.9*
CXCL9 (MIG)	384.3		350.8		*0.9*		1577.8		*4.1*		4573.7		*11.9*		295.8		*0.8*
CXCL10 (IP-10)	157.1		281.3		*1.8*		1209.8		*7.7*		1443.6		*9.2*		220.9		*1.4*
CSF3 (G-CSF)	332.9		640.6		*1.9*		7728.0		*23.2*		38436.3		*115.5*		1145.6		*3.4*
GM-CSF	14.7		18.4		*1.3*		26.2		*1.8*		60.5		*4.1*		11.0		*0.8*
IFN gamma	2.8		2.8		*1.0*		4.1		*1.5*		30.9		*11.0*		2.5		*0.9*
IL-1 alpha	126.1		292.8		*2.3*		264.1		*2.1*		430.1		*3.4*		78.7		*0.6*
IL-1 beta	3.6		4.6		*1.3*		8.7		*2.4*		17.0		*4.7*		2.8		*0.8*
IL-2	3.2		3.2		*1.0*		3.2		*1.0*		3.2		*1.0*		3.2		*1.0*
IL-4	3.0		3.0		*1.0*		3.0		*1.0*		3.0		*1.0*		3.0		*1.0*
IL-5	10.5		31.9		*3.0*		32.4		*3.1*		47.1		*4.5*		54.0		*5.1*
IL-6	6.8		54.4		*8.0*		169.8		*24.9*		632.0		*92.5*		55.5		*8.1*
IL-7	3.0		3.7		*1.2*		3.7		*1.2*		3.0		*1.0*		3.0		*1.0*
IL-10	2.5		3.5		*1.4*		44.9		*17.8*		36.4		*14.5*		3.3		*1.3*
IL-12p70	2.7		4.4		*1.6*		5.3		*1.9*		17.4		*6.3*		2.7		*1.0*
IL-13	24.9		89.1		*3.6*		96.0		*3.9*		178.9		*7.2*		53.7		*2.2*
IL-15	17.2		53.3		*3.1*		37.6		*2.2*		26.3		*1.5*		17.2		*1.0*
TNF alpha	3.1		8.3		*2.7*		22.4		*7.3*		23.7		*7.7*		4.3		*1.4*

## Discussion

An adjuvant is expected to promote adaptive immune responses to co-delivered antigens, with modulation of innate immunity in the local tissue environment as a key mechanism of activity. In this study, adjuvant responses at different time points following treatment were evaluated by several methods including gene transcriptional responses, cytokine expression, inflammatory responses in local tissues, and cellular responses. Both GLA-containing adjuvant formulations induced strong expression of MyD88 and NFκB dependent transcripts reflective of TLR4 signaling. Overall, the magnitude of transcriptional activation at the injection site, serum cytokine expression, and leukocyte activation was more enhanced by GLA-SE compared to GLA. The duration of transcriptional activation and serum cytokine expression was also enhanced with GLA-SE compared to GLA. This provides a molecular rationale for the enhanced adjuvant potency of the oil-in-water GLA-SE formulation compared to the aqueous GLA formulation [5]. In contrast, alum and SE did not induce transcriptional profiles or cytokine induction as robustly as either GLA-SE or GLA.

In this study, GLA rather than MPL was chosen as a representative TLR4 agonist adjuvant to compare the effects of formulation on innate *in vivo* activity. MPL is a heterogeneous mixture of detoxified lipopolysaccharide species purified from *Salmonella minnesota* [15]. The Lipid A components of these species contain 4–7 variable-length lipid chains attached to the polysaccharide backbone, and these different species can have different relative potency [16]. GLA is produced synthetically such that the Lipid A component contains exactly 6 acyl lipid chains of uniform length bound to the polysaccharide backbone [15]. In aqueous formulations, GLA and MPL were compared and found to similarly induce gene expression and cytokines in mouse and human dendritic cells *in vitro* [9], [17]. Additionally, GLA and MPL in emulsion formulations were compared and found to have similar activity in the induction of serum cytokine proteins *in vivo* [9].

TLR4 agonist adjuvants such as GLA and GLA-SE have been reported to induce T_H_1-type cytokine responses to coadministered antigens and boost antigen-specific IgG2a responses [5]. Innate cytokines play a major role in gearing the adaptive immune response towards T_H_1 or T_H_2 [18]. The role of TLR4 in inducing innate cytokines associated with T_H_1 responses has been previously demonstrated by *in vitro* studies [9]. In this *in vivo* study, we found that GLA and GLA-SE strongly induced chemokine transcripts (CXCL9, CXCL10, and CXCL11) that could recruit activated T_H_1 T cells to the site of injection. Consistent with this, increased serum levels of CXCL9 and CXCL10 were also detected. In the dLN, activated T_H_1 T cell transcripts including IFNγ, STAT1 and CCR5 transcripts were increased following GLA-SE treatment. In response to GLA or GLA-SE, dLN had enhanced numbers of activated DC and enhanced MHC I and MHC II expression on activated DC. These data suggest a molecular mechanism of action for the *in vivo* T_H_1 bias observed with GLA and GLA-SE with both capable of enhancing antigen presentation that could stimulate cellular immune responses to coadministered antigens. In contrast, the T_H_2-biasing adjuvants alum and SE did not induce CXCL9, CXCL10, or IFNγ at the gene or protein level, and rather induced the T_H_2 cytokine IL-5 in serum.

Various formulations of alum adjuvant have been used in vaccine studies. Salts of both Al(OH)_3_ and Al(PO_4_) are referred to as “alum” and are available from multiple manufacturers. In this study, Alhydrogel obtained from Brenntag generated enhanced serum levels of CCL2, CCL3, CSF3, IL-6 and TNFα, consistent with a previous report that Alhydrogel increased expression of these proteins at the site of injection [8]. We also observed 2-fold upregulated serum IL-1α, corresponding to alum's ability to induce enhanced processing of pro-forms of IL-1 family members through NALP3-mediated caspase activity [19]. However, we did not see significant transcriptional activation of these cytokines at the injection site although alum obtained from Pierce was reported to do so [7]. The differences observed between our study and [7] may be due to the use of different sources of alum.

The oil-in-water emulsion SE has not been evaluated previously for its molecular mechanism of action. We found that CCL2, CCL6, CXCL1 and CXCL10 transcripts induced by SE at the injection site were more than 2-fold higher than the control treatment at 24 hours. Higher levels of CCL2, CXCL1, CSF3, IL-5 and IL-6 proteins were also detected in serum. Other oil-in-water emulsions have been reported to have similar activity. AS03 was reported to induce transcriptional upregulation of CCL3, CCL4, CXCL1, IL-6, and CSF3 in muscle and dLN by 24 hours [8]. MF59 was found to induce transcription of CCL2, CCL3, CCL6, CXCL10, IL-6 and CSF1 in the muscle within 24 hours [7]. Interestingly, the SE used in our study appeared to induce fewer genes than that reported for either MF59 or AS03, suggesting that different squalene-based oil-in-water emulsions may have slightly different immunostimulatory effects.

Immunostimulatory gene transcription induction by adjuvant compounds both at the local site of injection and in draining lymph nodes can promote migration of responding cells. Small particles (<200 nm) such as the adjuvants tested here traffic quickly through lymphatics to lymph nodes following injection into muscle [20]. In local muscle tissues, pro-inflammatory cytokine transcripts such as IL-6, TNFα, IL-1α, and IL-1β and monocyte chemoattractant transcripts such as CCL2, CXCL1, and CSF3 were found upregulated by GLA-SE at early timepoints. These transcripts and those for immature DC chemoattractants such as CCL3 and CCL4 were also upregulated in dLN. In concordance with this finding, by 24 hours following GLA and GLA-SE treatment a large influx of cells was found in dLN, such as increased numbers of CD11c+ dendritic cells and T lymphocytes. MHC I molecules are known to be upregulated by pro-inflammatory cytokines and IFNγ [21]. As expected, upregulated H2-D1 and H2-K1 MHC I transcripts were found in dLN and increased surface expression of MHC I was found on CD11c+ dendritic cells in dLN following GLA-SE treatment. The increased numbers of T lymphocytes found in dLN following GLA-SE treatment corresponded with the high transcription of T cell chemokines CXCL9, CXCL11 and IFNγ induced in dLN following GLA-SE treatment. While B cells in dLN weren't evaluated, high dLN expression of the B cell chemokine CXCL13 suggests that B cells may also be selectively recruited to dLN.

The limited and short-term upregulation of gene transcripts detected in blood following GLA-SE treatment suggest that this adjuvant has the localized, transient response associated with a favorable safety profile in humans [22]. IL-6, TNFα, and IL-1β are the cytokines associated with acute phase responses when present at high levels in blood [23]. Each of these cytokines plays an important role in the induction of adaptive immunity, with IL-6 particularly important to T cell activation [24]. IL-6, TNFα, and IL-1β transcripts were increased by GLA-SE in muscle and dLN, but not in blood. Despite upregulated IL-6, TNFα, and IL-1β transcripts in muscle and dLN, serum cytokines corresponding to these transcripts were transient, peaking at 6 hours and nearly undetectable by 24 hours (Figure 5). Circulating serum amyloid A (SAA) is produced in response to IL-6, IL-1β, and TNFα by adipocytes and macrophages and is reflective of acute phase reactions [25]. Saa1, Saa2, and Saa3 transcripts were detected in muscle (data not shown), but only Saa3 transcripts were detected in blood. Saa3 transcripts in blood peaked at 6 hours (6.8 fold induced) and declined to baseline by 48 hours. This limited and short-term expression of Saa1 and Saa2 is consistent with a transiently localized inflammatory response. We speculate that regulatory mechanisms may limit translation of these transcripts and that proteins induced by the adjuvant could be taken up by cells near the site of production to prohibit their wide diffusion.

Measurement of early innate immune responses to vaccines may supplement the evaluation of induced adaptive immune responses in determining the appropriate dosing of an adjuvant. This may be of particular interest in target populations known to have deficiencies in innate responses, such as the elderly. Monitoring innate responses could identify an adjuvant dose and formulation that achieves the desired immunostimulation profile. In this study, the potency of a TLR4 adjuvant as measured by induced cytokines could be modulated both by adjuvant dose and by the choice of formulation (GLA versus GLA-SE).

In conclusion, this study has demonstrated that GLA-SE adjuvant induces multiple transcriptional and translational responses detectable at the site of injection, in dLN and in peripheral blood. The GLA-dependent induction of chemokines specific for lymphocytes and antigen presenting cells and the enhanced levels of dendritic cell activation in the draining lymph nodes provide strong molecular and cellular basis for the mechanism of action of GLA-SE adjuvant in induction of T_H_1 biased cellular and humoral responses to coadministered antigens. Formulation of GLA as GLA-SE increased the innate potency of the adjuvant, allowing dose-sparing, and extended the duration of the innate response. The understanding of the role of TLR4 agonist pathways in promoting appropriate vaccine responses is critical to designing vaccines with appropriate immune responses.

## Materials and Methods

### Adjuvants

Alhydrogel alum (aluminum hydroxide) was obtained from Accurate Chemical & Scientific (Westbury, NY). GLA, GLA-SE, and SE were obtained from Immune Design Corporation (Seattle WA) [15].

### Animals

Female BALB/c mice (7–10 weeks old) were purchased from Charles River Laboratories (Hollister, CA) and housed under pathogen-free conditions in the animal facility at MedImmune. All animal work was performed on protocol 078 in accordance with the MedImmune International Animal Care and Use Committee policies.

### Study Design

Mice temporarily anesthetized with isoflorane were intramuscularly injected with PBS, alum (100 µg), GLA (5 µg), SE (2%), or GLA-SE (5 µg/2%) in a 100 μl volume with 50 μl given per quadriceps. Quadriceps muscles, draining lymph nodes (both iliac and inguinal), whole blood and serum samples were obtained from treatment cohorts of 3 mice per group at each of the indicated timepoints 6 hours, 24 hours, 48 hours, and 96 hours after treatment. Two or more replicate experiments were run for each analysis conducted. Muscle tissues from the injection site were collected, cut into pieces (<0.5 cm) and stored in RNAlater (Qiagen, Valencia, CA) at −80°C for RNA extraction. A segment of muscle from each animal was stored in 10% buffered formalin at room temperature for histological evaluation. Tissue sections were stained with H & E and evaluated by a pathologist. The draining lymph nodes (iliac and popliteal) were harvested into RNAlater at −80°C for RNA extraction or mechanically dispersed in RPMI to create single cell suspensions prior to antibody staining. For the whole blood (WB) samples, 0.5 ml was collected using 2-ml Qiagen RNAprotect animal blood tubes (Qiagen, Valencia, CA) and stored at −80°C. Sera were collected using BD Microtainer® tubes which contained clot activator reagents (BD, Franklin Lakes, NJ).

### RNA extraction

Approximately 15–20 mg of muscle or lymph node tissues from each individual animal was homogenized using Tissue Lyser (Qiagen, Valencia, CA) by pulsing for 2 minutes at 20 Hz twice. RNA was subsequently extracted from the tissue homogenates using RNeasy mini kit according to manufacturer's protocol (Qiagen, Valencia, CA), and total RNA was eluted using 30 µl of RNase-free water. WB RNA was extracted using a silica-membrane based RNAeasy protect animal blood kit following the manufacturer's procedures (Qiagen, Valencia, CA) and an on-column DNase I treatment was included. Mouse globin mRNA was carefully removed from the total WB RNA by hybridization to mouse globin-specific oligonucleotides which was captured by magnetic beads through biotin and streptavidin binding (Ambion, Austin, TX). From a 0.5 ml WB, a final 30 µl of globin-cleared blood RNA was generated. The quality and quantity of all RNA samples were assessed by Agilent Bioanalyzer 2100 (Agilent, Santa Clara, CA) and NanoDrop 2000 (Thermo Fisher Scientific, Auburn, AL), respectively.

### Microarray processing and data analysis

Total RNA was labeled and fragmented according to the Affymetrix GeneChip® 3′ IVT express kit procedure (Affymetrix, Santa Clara, CA). In brief, 400 ng of total RNA was used to generate 1^st^ strand cDNA by using Superscript III reverse transcriptase with a T7-oligo (dT) primer. Subsequently, the 2^nd^ strand cDNA synthesis was carried out using a combination of DNA polymerase I and *E. coli* DNA ligase. The resulting cDNA was purified and biotin-labeled using T7 RNA polymerase and biotinylated ribonucleotides, and was fragmented by metal-induced hydrolysis. The labeled, fragmented complementary RNA of 12.5 μg was hybridized to the Affymetrix GeneChip Mouse Genome 430 2.0 Array for 16 hours at 45°C. After washing and staining, the GeneChip arrays were scanned and the images were processed using Affymetrix software, GeneChip Operating Software (GCOS). For each run, individual replicates of three mice per timepoint and per treatment group were used, and each sample replicate was run on duplicate chips. There was a correlation of greater than 90% between the two chips for each sample, and a P-value less than 0.05 for the replicates.

Raw microarray data were analyzed using the R programming language. Raw data were normalized by an RMA algorithm method, and differentially expressed gene probe sets were identified using the Limma package of software, where a standard T test was performed between treatment and control groups, to derive fold changes, p-values, and directions of regulation for each probe-set. Differentially expressed gene probe sets were defined as a fold change versus PBS control of greater than 2 with a p-value of less than 0.05. The identified probe sets were further compared using GeneSpring software (GX).

### Histological examination of muscle injection site

Muscle sections (4 micron) were prepared from paraffin-embedded formalin-fixed tissue using a microtome and stained with hematoxylin and eosin. Histopathological changes such as cellular infiltrates and myocyte alterations were evaluated by a board-certified pathologist and scored as to distribution and severity.

### Flow cytometry analysis

Red blood cell depleted lymph node single cell suspensions were distributed in 96-well microtiter plates at 1×10^6^ cells/well. Cells were stained for viability with LIVE/DEAD Fixable Blue (Invitrogen Life Technologies, Grand Island, NY), followed by CD3-AlexaFluor700, GR1-PerCP-Cy5, CD11c-PE-Cy7, CD54-PE, and either I-A^d^-FITC or H-2k^d^-FITC (all from BD Biosciences, San Jose, CA). Following overnight fixation with 2% paraformaldehyde, 300,000–500,000 events were aquired on a LSR 2 (BD Biosciences), and data was analyzed using Diva software (BD Biosciences).

### Serum Cytokine Analysis

Serum was separated by centrifugation at 10,000 rpm for 8 minutes at room temperature and stored at −80°C until evaluation. Bead-based custom cytokine multiplex immunoassay kits from Millipore (Billerica, MA) including G-CSF (CSF3), IFNγ, IL-5, IL-6, IP-10 (CXCL10), KC (CXCL1), MCP-1 (CCL2), TNFα and other cytokines were used according to the manufacturer's protocol. Data was acquired on the BioRad Bio-Plex 200 reader (Hercules, CA) and analyzed using Bioplex software.

### Statistics

Data was analyzed using Prism GraphPad software. Data shown is representative of two or more experiments. All data is expressed as arithmetic mean ± standard error of the mean (SEM). Statistical significance was calculated by one way ANOVA followed by a Tukey post test with a cutoff of p<0.05.

## Supporting Information

Table S1 (DOC)

Table S2 (DOC)
