# Supplementary material for: Molecular and Cellular Response Profiles Induced by the TLR4 Agonist-Based Adjuvant Glucopyranosyl Lipid A
Source: PLoS One. 2012 Dec 28;7(12):e51618. doi: 10.1371/journal.pone.0051618 (PMC3532059; doi:10.1371/journal.pone.0051618)
Supplement: Table S1 — (DOC) [file pone.0051618.s001.doc]

**Table S1 Selected genes upregulated in muscle post adjuvant, fold-change from PBS**

| **MyD88 Dependent Genes** | | | | | | | | | | | | | | | | | |
| --- | --- | --- | --- | --- | --- | --- | --- | --- | --- | --- | --- | --- | --- | --- | --- | --- | --- |
|  | **Alum** | | | | | **GLA** | | | | **GLA-SE** | | | | **SE** | | | |
| **Symbol** | **6h** | | **24h** | **48h** | **96h** | **6h** | **24h** | **48h** | **96h** | **6h** | **24h** | **48h** | **96h** | **6h** | **24h** | **48h** | **96h** |
| **IRAK2** | 1.1 | | 1.1 | 1.1 | 1.0 | 2.0 | 1.1 | 1.4 | 0.8 | 2.3 | 2.5 | 1.9 | 1.3 | 1.2 | 0.9 | 0.9 | 3.6 |
| **MYD88** | 0.9 | | 1.2 | 0.9 | 1.0 | 5.8 | 1.8 | 1.4 | 0.9 | 8.0 | 8.9 | 6.4 | 2.5 | 1.1 | 1.1 | 1.0 | 3.9 |
| **NR2C2** | 1.0 | | 1.0 | 1.0 | 1.0 | 0.9 | 1.2 | 1.0 | 1.2 | 0.9 | 1.2 | 0.9 | 1.2 | 0.9 | 1.0 | 0.8 | 0.9 |
| **TIRAP** | 0.9 | | 1.0 | 1.0 | 1.1 | 1.3 | 0.8 | 1.1 | 1.4 | 0.9 | 0.7 | 0.6 | 1.2 | 1.8 | 0.5 | 0.6 | 1.3 |
| **TLR1** | 1.1 | | 1.2 | 1.0 | 1.0 | 6.0 | 3.8 | 4.2 | 1.3 | 2.7 | 6.6 | 47.9 | 18.5 | 1.0 | 1.6 | 1.1 | 2.4 |
| **TLR2** | 0.8 | | 1.1 | 1.1 | 0.8 | 6.9 | 1.7 | 1.9 | 1.0 | 6.8 | 5.2 | 8.1 | 8.4 | 1.0 | 1.2 | 1.0 | 1.6 |
| **TLR5** | 0.9 | | 0.9 | 0.9 | 1.0 | 0.9 | 1.0 | 1.1 | 1.3 | 0.9 | 0.9 | 0.9 | 1.1 | 1.0 | 1.0 | 1.0 | 1.2 |
| **TLR6** | 1.1 | | 1.0 | 0.9 | 0.9 | 1.6 | 1.0 | 0.9 | 1.0 | 1.5 | 1.3 | 1.8 | 1.2 | 1.0 | 0.9 | 1.0 | 2.3 |
| **TLR7** | 0.9 | | 1.0 | 1.0 | 1.0 | 1.0 | 1.1 | 1.2 | 1.2 | 1.1 | 1.5 | 2.2 | 4.6 | 1.0 | 0.8 | 0.9 | 1.0 |
| **TLR8** | 0.8 | | 1.0 | 0.8 | 1.0 | 0.9 | 1.2 | 1.1 | 1.2 | 0.8 | 1.1 | 0.9 | 0.9 | 1.1 | 0.8 | 0.9 | 0.9 |
| **TLR9** | 0.7 | | 1.0 | 0.9 | 1.0 | 0.9 | 1.0 | 1.0 | 1.4 | 0.9 | 1.0 | 0.9 | 1.2 | 1.0 | 0.9 | 1.1 | 0.8 |
| **TRIF Dependent Genes** | | | | | | | | | | | | | | | | | |
|  | **Alum** | | | | | **GLA** | | | | **GLA-SE** | | | | **SE** | | | |
| **Symbol** | **6h** | | **24h** | **48h** | **96h** | **6h** | **24h** | **48h** | **96h** | **6h** | **24h** | **48h** | **96h** | **6h** | **24h** | **48h** | **96h** |
| **GBP2** | 1.0 | | 1.1 | 1.0 | 0.9 | 18.8 | 7.7 | 1.8 | 1.4 | 19.9 | 44.4 | 13.5 | 2.5 | 1.2 | 1.4 | 1.2 | 7.1 |
| **IFIT1** | 0.8 | | 0.9 | 1.3 | 1.6 | 106.1 | 9.0 | 2.2 | 1.0 | 59.7 | 29.0 | 44.6 | 11.2 | 1.4 | 2.0 | 1.4 | 9.1 |
| **IFIT2** | 1.2 | | 1.0 | 1.2 | 1.1 | 49.1 | 5.2 | 1.4 | 0.8 | 32.8 | 14.7 | 15.6 | 8.6 | 1.1 | 1.3 | 1.0 | 4.4 |
| **IFIT3** | 0.9 | | 0.8 | 1.1 | 1.2 | 45.6 | 5.8 | 1.8 | 1.0 | 22.6 | 9.7 | 16.2 | 5.5 | 1.1 | 1.5 | 1.2 | 2.2 |
| **IRF7** | 1.0 | | 1.0 | 1.1 | 1.2 | 9.9 | 8.3 | 4.0 | 2.0 | 9.1 | 17.9 | 23.9 | 12.9 | 1.2 | 1.6 | 1.3 | 3.0 |
| **MX1** | 1.0 | | 1.1 | 1.1 | 1.1 | 26.7 | 1.4 | 1.1 | 1.0 | 12.4 | 6.1 | 10.4 | 2.9 | 0.8 | 1.1 | 0.8 | 2.4 |
| **OASL1** | 0.8 | | 0.9 | 1.0 | 0.9 | 18.0 | 1.6 | 1.8 | 1.2 | 15.0 | 8.9 | 18.3 | 5.2 | 1.1 | 1.2 | 1.2 | 3.7 |
| **OASL2** | 1.2 | | 1.0 | 1.2 | 1.3 | 25.1 | 9.1 | 2.0 | 1.2 | 22.3 | 21.8 | 15.5 | 10.3 | 1.2 | 2.4 | 1.4 | 1.9 |
| **SOCS3** | 0.7 | | 0.8 | 1.1 | 1.4 | 21.0 | 3.0 | 2.3 | 2.0 | 42.3 | 45.8 | 7.8 | 3.2 | 0.6 | 2.9 | 0.5 | 1.2 |
| **STAT5A** | 1.2 | | 1.1 | 1.2 | 0.8 | 2.1 | 0.9 | 0.9 | 0.8 | 1.6 | 0.8 | 1.0 | 0.7 | 0.9 | 0.9 | 1.1 | 1.7 |
| **TLR3** | 1.0 | | 1.2 | 0.9 | 1.0 | 13.1 | 1.4 | 1.3 | 0.8 | 8.7 | 3.2 | 3.7 | 2.0 | 1.2 | 1.3 | 1.1 | 5.6 |
| **Cytokines/Chemokine Genes** | | | | | | | | | | | | | | | | | |
|  | **Alum** | | | | | **GLA** | | | | **GLA-SE** | | | | **SE** | | | |
| **Symbol** | **6h** | | **24h** | **48h** | **96h** | **6h** | **24h** | **48h** | **96h** | **6h** | **24h** | **48h** | **96h** | **6h** | **24h** | **48h** | **96h** |
| **CCL1** | 0.8 | | 1.0 | 1.0 | 0.9 | 0.8 | 1.0 | 0.9 | 0.9 | 1.0 | 1.0 | 0.9 | 0.8 | 1.0 | 0.9 | 1.1 | 0.8 |
| **CCL2** | 0.7 | | 0.5 | 1.0 | 1.0 | 54.6 | 2.4 | 2.6 | 1.0 | 56.4 | 35.6 | 34.2 | 6.0 | 1.0 | 2.6 | 1.0 | 1.1 |
| **CCL3** | 1.0 | | 1.1 | 0.9 | 1.0 | 19.5 | 1.4 | 1.1 | 1.1 | 44.7 | 19.3 | 14.0 | 3.8 | 1.1 | 1.1 | 1.0 | 1.0 |
| **CCL4** | 0.9 | | 0.9 | 0.9 | 0.9 | 6.2 | 1.4 | 1.1 | 1.4 | 11.5 | 5.0 | 4.4 | 1.5 | 1.0 | 0.8 | 1.0 | 1.0 |
| **CCL5** | 0.9 | | 1.2 | 0.9 | 0.9 | 36.1 | 7.6 | 3.2 | 2.3 | 18.5 | 24.7 | 26.3 | 20.2 | 1.1 | 1.2 | 1.1 | 4.2 |
| **CCL6** | 1.1 | | 1.6 | 1.0 | 1.9 | 3.4 | 7.6 | 8.3 | 2.0 | 1.2 | 5.2 | 23.4 | 48.7 | 1.7 | 9.2 | 1.3 | 6.4 |
| **CCL7** | 0.6 | | 0.6 | 1.0 | 1.1 | 18.9 | 1.4 | 1.7 | 1.4 | 16.0 | 20.1 | 26.2 | 3.9 | 0.9 | 2.7 | 0.9 | 1.1 |
| **CCL9** | 0.8 | | 1.1 | 1.1 | 2.0 | 7.6 | 5.6 | 7.0 | 1.6 | 2.3 | 4.4 | 26.0 | 33.5 | 1.0 | 7.4 | 1.1 | 3.1 |
| **CCL11** | 1.2 | | 0.8 | 0.9 | 1.6 | 2.6 | 1.3 | 1.1 | 1.3 | 4.0 | 1.8 | 1.3 | 3.8 | 1.7 | 2.3 | 1.3 | 1.6 |
| **CSF3** | 0.8 | | 1.1 | 0.9 | 1.0 | 3.4 | 1.2 | 0.9 | 1.2 | 21.4 | 2.7 | 1.0 | 0.9 | 1.0 | 0.8 | 0.9 | 0.9 |
| **CXCL1** | 0.7 | | 0.9 | 1.1 | 1.2 | 81.9 | 3.0 | 1.2 | 1.2 | 235.2 | 40.6 | 17.3 | 1.9 | 1.0 | 1.8 | 1.1 | 1.1 |
| **CXCL2** | 1.0 | | 0.9 | 1.1 | 0.9 | 58.4 | 1.4 | 1.2 | 0.9 | 116.3 | 26.0 | 10.8 | 1.8 | 0.9 | 0.9 | 0.9 | 1.0 |
| **CXCL5** | 0.6 | | 0.8 | 1.0 | 0.9 | 23.2 | 0.9 | 1.1 | 0.9 | 95.4 | 23.3 | 22.3 | 1.1 | 1.1 | 1.0 | 0.9 | 0.9 |
| **CXCL9** | 1.2 | | 1.5 | 1.1 | 0.9 | 116.3 | 33.6 | 3.6 | 3.9 | 94.1 | 310.9 | 66.9 | 5.5 | 1.2 | 1.2 | 1.0 | 27.1 |
| **CXCL10** | 1.0 | | 0.9 | 1.0 | 0.9 | 227.3 | 8.6 | 1.3 | 0.9 | 175.5 | 79.4 | 28.2 | 1.8 | 1.0 | 1.4 | 1.1 | 1.7 |
| **CXCL11** | 1.1 | | 0.8 | 0.8 | 1.0 | 21.5 | 4.2 | 1.0 | 1.0 | 31.9 | 28.4 | 5.3 | 1.2 | 0.9 | 0.8 | 0.9 | 1.1 |
| **CXCL13** | 1.0 | | 0.4 | 1.1 | 1.1 | 21.4 | 21.5 | 12.3 | 2.0 | 3.8 | 44.4 | 41.1 | 2.2 | 0.9 | 1.3 | 0.9 | 10.9 |
| **IFNG** | 1.0 | | 1.0 | 0.9 | 0.9 | 1.0 | 1.1 | 0.9 | 0.9 | 1.3 | 1.2 | 1.2 | 0.9 | 1.1 | 1.0 | 1.1 | 1.0 |
| **IL1A** | 0.9 | | 1.0 | 1.0 | 1.0 | 1.4 | 1.0 | 1.0 | 0.9 | 1.3 | 1.4 | 1.0 | 1.0 | 1.0 | 1.0 | 1.0 | 1.0 |
| **IL1B** | 0.8 | | 1.0 | 1.1 | 1.1 | 25.4 | 1.5 | 1.4 | 1.1 | 24.0 | 24.8 | 18.0 | 2.3 | 1.1 | 1.1 | 1.2 | 1.2 |
| **IL1RN** | 0.9 | | 0.9 | 1.0 | 0.8 | 46.0 | 1.2 | 1.2 | 0.8 | 38.2 | 15.1 | 9.0 | 2.6 | 1.1 | 1.3 | 1.0 | 1.1 |
| **IL5** | 0.9 | | 1.0 | 0.9 | 1.2 | 1.0 | 1.1 | 0.9 | 1.1 | 1.0 | 1.1 | 0.8 | 0.9 | 1.0 | 1.0 | 1.0 | 1.0 |
| **IL6** | 1.0 | | 1.0 | 0.9 | 1.0 | 64.0 | 1.2 | 0.9 | 1.1 | 168.5 | 57.9 | 6.3 | 1.0 | 1.1 | 1.0 | 1.0 | 1.0 |
| **IL10** | 1.0 | | 1.0 | 1.0 | 0.9 | 1.0 | 1.1 | 1.1 | 1.0 | 0.9 | 1.0 | 1.1 | 1.0 | 0.9 | 1.0 | 1.1 | 0.9 |
| **IL18** | 1.0 | | 1.0 | 1.1 | 1.0 | 1.0 | 1.0 | 1.1 | 0.9 | 11.0 | 1.0 | 1.0 | 0.9 | 6.5 | 1.4 | 2.2 | 1.5 |
| **SPP1** | 0.5 | | 0.6 | 0.7 | 0.7 | 3.5 | 1.1 | 2.1 | 0.8 | 2.9 | 2.2 | 4.1 | 3.3 | 1.0 | 1.0 | 0.4 | 3.7 |
| **TNF** | 1.0 | | 0.9 | 0.8 | 1.0 | 2.1 | 1.1 | 0.9 | 1.2 | 2.2 | 2.3 | 2.1 | 1.1 | 1.0 | 1.1 | 1.0 | 1.0 |
| **Cytokine Receptors and Signaling Molecule Genes** | | | | | | | | | | | | | | | | | |
|  | | **Alum** | | | | **GLA** | | | | **GLA-SE** | | | | **SE** | | | |
| **Symbol** | | **6h** | **24h** | **48h** | **96h** | **6h** | **24h** | **48h** | **96h** | **6h** | **24h** | **48h** | **96h** | **6h** | **24h** | **48h** | **96h** |
| **CCR1** | | 1.0 | 1.0 | 1.0 | 1.1 | 9.5 | 1.5 | 1.7 | 0.9 | 5.5 | 6.1 | 14.1 | 2.0 | 1.1 | 1.7 | 1.0 | 2.0 |
| **CCR2** | | 1.1 | 0.9 | 1.0 | 0.9 | 6.7 | 4.5 | 10.6 | 1.5 | 3.0 | 3.1 | 26.2 | 22.9 | 0.9 | 1.4 | 0.9 | 3.8 |
| **CCR5** | | 1.2 | 0.9 | 0.9 | 1.1 | 8.8 | 2.0 | 5.1 | 1.2 | 3.3 | 5.3 | 28.7 | 26.4 | 1.0 | 1.6 | 0.9 | 2.9 |
| **CSF1R** | | 1.7 | 1.2 | 1.0 | 1.1 | 1.9 | 1.3 | 3.4 | 1.0 | 1.0 | 1.6 | 5.1 | 14.2 | 0.8 | 2.0 | 1.0 | 9.8 |
| **IFNAR2** | | 1.2 | 1.4 | 1.2 | 0.8 | 5.4 | 1.4 | 1.8 | 0.9 | 6.3 | 4.1 | 8.2 | 5.5 | 0.9 | 1.3 | 1.0 | 5.8 |
| **IFNGR1** | | 1.1 | 1.1 | 1.1 | 0.9 | 2.5 | 1.3 | 1.3 | 1.0 | 2.1 | 1.6 | 1.6 | 2.2 | 1.2 | 1.3 | 0.9 | 2.9 |
| **IL1R2** | | 0.8 | 1.1 | 0.8 | 0.8 | 3.5 | 1.3 | 0.9 | 0.9 | 7.1 | 2.8 | 1.5 | 0.9 | 1.2 | 1.0 | 1.0 | 2.0 |
| **IL6RA** | | 1.3 | 1.6 | 1.0 | 1.1 | 1.6 | 2.6 | 2.6 | 0.8 | 5.4 | 10.2 | 5.3 | 6.1 | 1.7 | 1.3 | 0.9 | 6.6 |
| **IL10RA** | | 1.0 | 1.0 | 0.9 | 1.1 | 1.3 | 1.0 | 1.1 | 1.3 | 1.2 | 1.5 | 3.6 | 4.9 | 1.0 | 1.3 | 1.1 | 1.0 |
| **IL10RB** | | 1.0 | 1.1 | 1.2 | 1.1 | 2.7 | 1.1 | 1.7 | 0.8 | 2.0 | 1.3 | 3.4 | 4.4 | 0.8 | 1.1 | 0.8 | 5.0 |
| **IL13RA** | | 0.7 | 0.9 | 1.2 | 1.2 | 4.0 | 1.4 | 1.4 | 1.2 | 6.8 | 4.0 | 3.3 | 4.4 | 0.9 | 1.2 | 0.9 | 1.6 |
| **IL15RA** | | 1.3 | 1.1 | 1.2 | 1.0 | 1.5 | 1.0 | 0.9 | 0.9 | 2.5 | 3.9 | 1.0 | 1.0 | 1.1 | 0.8 | 1.0 | 0.9 |
| **STAT1** | | 1.0 | 0.8 | 1.1 | 1.3 | 8.3 | 4.6 | 2.2 | 1.3 | 3.9 | 20.4 | 28.1 | 6.0 | 1.1 | 1.3 | 1.2 | 5.4 |
| **STAT3** | | 0.7 | 1.0 | 1.1 | 1.2 | 1.4 | 1.7 | 1.2 | 1.3 | 2.2 | 5.0 | 2.0 | 1.6 | 1.2 | 1.4 | 0.9 | 1.3 |
| **TNFRSF1B** | | 1.0 | 1.1 | 0.8 | 1.0 | 4.8 | 1.9 | 3.3 | 1.1 | 4.6 | 5.8 | 13.7 | 11.1 | 1.1 | 1.1 | 1.1 | 2.7 |
| **Complement and Antigen Processing/Presentation Genes** | | | | | | | | | | | | | | | | | |
|  | **Alum** | | | | | **GLA** | | | | **GLA-SE** | | | | **SE** | | | |
| **Symbol** | **6h** | | **24h** | **48h** | **96h** | **6h** | **24h** | **48h** | **96h** | **6h** | **24h** | **48h** | **96h** | **6h** | **24h** | **48h** | **96h** |
| **B2M** | 1.1 | | 1.1 | 1.2 | 1.2 | 4.8 | 2.5 | 3.0 | 2.1 | 3.2 | 5.2 | 5.6 | 8.2 | 1.1 | 1.5 | 1.0 | 10.1 |
| **C1QA** | 1.3 | | 1.2 | 1.1 | 2.0 | 2.6 | 1.9 | 3.3 | 2.4 | 0.5 | 1.5 | 2.9 | 21.4 | 0.9 | 3.0 | 0.9 | 9.4 |
| **C1QB** | 1.4 | | 1.4 | 0.9 | 1.4 | 6.5 | 2.3 | 3.9 | 2.2 | 1.3 | 2.2 | 5.2 | 27.7 | 0.9 | 3.5 | 0.8 | 11.0 |
| **C1QC** | 1.2 | | 1.3 | 1.1 | 1.8 | 2.5 | 1.8 | 3.6 | 1.6 | 0.6 | 1.5 | 3.5 | 17.7 | 0.8 | 2.8 | 0.9 | 8.9 |
| **C3** | 1.1 | | 2.3 | 1.1 | 0.9 | 7.8 | 5.1 | 8.0 | 2.3 | 4.6 | 10.8 | 9.4 | 9.7 | 1.0 | 4.3 | 1.2 | 27.8 |
| **FCER1G** | 1.0 | | 1.2 | 1.0 | 1.4 | 6.7 | 4.3 | 7.1 | 1.9 | 4.8 | 10.1 | 32.6 | 34.3 | 0.9 | 2.4 | 0.9 | 6.2 |
| **FCGR1** | 1.0 | | 0.7 | 1.1 | 1.0 | 9.4 | 2.1 | 3.1 | 1.1 | 3.2 | 7.1 | 45.4 | 16.5 | 0.8 | 1.6 | 0.8 | 1.4 |
| **H2-AA** | 1.4 | | 2.8 | 1.1 | 1.2 | 3.3 | 0.5 | 2.4 | 2.3 | 0.9 | 1.7 | 2.0 | 15.4 | 0.5 | 1.3 | 1.1 | 11.7 |
| **H2-D1** | 1.2 | | 1.2 | 1.4 | 0.7 | 5.8 | 2.6 | 4.2 | 1.5 | 3.6 | 6.1 | 10.2 | 8.4 | 1.2 | 1.5 | 0.8 | 20.0 |
| **H2-EA** | 1.4 | | 2.9 | 1.1 | 1.1 | 4.2 | 0.4 | 2.3 | 2.0 | 1.3 | 1.4 | 2.0 | 11.7 | 0.6 | 1.2 | 1.1 | 10.3 |
| **H2-K1** | 1.2 | | 1.4 | 1.4 | 0.8 | 6.0 | 3.6 | 4.1 | 1.5 | 5.2 | 6.9 | 11.3 | 8.8 | 1.2 | 1.3 | 1.1 | 17.8 |
| **TAP2** | 1.2 | | 1.1 | 1.3 | 0.9 | 8.7 | 2.6 | 1.4 | 1.3 | 6.5 | 10.4 | 3.9 | 1.8 | 1.2 | 1.3 | 1.1 | 8.1 |
| **TAPBP** | 1.2 | | 1.3 | 1.0 | 0.9 | 3.6 | 2.8 | 1.9 | 0.9 | 3.3 | 6.2 | 6.1 | 2.5 | 0.9 | 1.2 | 0.9 | 4.5 |
