# Supplementary material for: Molecular and Cellular Response Profiles Induced by the TLR4 Agonist-Based Adjuvant Glucopyranosyl Lipid A
Source: PLoS One. 2012 Dec 28;7(12):e51618. doi: 10.1371/journal.pone.0051618 (PMC3532059; doi:10.1371/journal.pone.0051618)
Supplement: Table S2 — (DOC) [file pone.0051618.s002.doc]

**Table S2 Selected GLA-SE induced genes in muscle, dLN, and blood, fold-change from PBS**

| **Cytokines/Chemokines** | | | | | | | | | |
| --- | --- | --- | --- | --- | --- | --- | --- | --- | --- |
|  | **6h** | | | **24h** | | | **48h** | | |
| **Symbol** | **Mus** | **dLN** | **blood** | **Mus** | **dLN** | **blood** | **Mus** | **dLN** | **blood** |
| **CCL1** | 1.1 | 1.0 | 0.8 | 1.0 | 1.1 | 1.1 | 0.9 | 1.0 | 1.0 |
| **CCL2** | 81.9 | 41.0 | 1.2 | 59.7 | 35.8 | 1.2 | 34.7 | 16.3 | 1.3 |
| **CCL3** | 43.6 | 115.9 | 0.9 | 19.1 | 13.4 | 0.6 | 14.9 | 7.9 | 0.8 |
| **CCL4** | 10.9 | 39.1 | 1.2 | 5.1 | 8.1 | 1.0 | 4.6 | 4.3 | 1.1 |
| **CCL5** | 21.3 | 1.2 | 0.7 | 26.4 | 0.9 | 0.2 | 24.5 | 1.1 | 0.4 |
| **CCL6** | 1.2 | 1.4 | 1.0 | 6.3 | 2.8 | 0.4 | 22.9 | 3.6 | 0.5 |
| **CCL7** | 21.8 | 28.0 | 1.1 | 31.6 | 21.5 | 1.6 | 26.2 | 8.3 | 1.1 |
| **CCL9** | 2.5 | 3.7 | 1.0 | 5.9 | 1.6 | 0.6 | 24.7 | 3.2 | 0.9 |
| **CCL11** | 4.0 | 7.2 | 1.0 | 1.5 | 1.4 | 1.2 | 1.1 | 1.3 | 1.2 |
| **CSF3** | 22.2 | 20.3 | 1.1 | 2.8 | 4.3 | 1.0 | 1.0 | 1.7 | 1.0 |
| **CXCL1** | 347.2 | 61.5 | 0.8 | 41.2 | 17.2 | 1.0 | 17.1 | 8.1 | 1.0 |
| **CXCL2** | 120.2 | 111.9 | 3.1 | 28.1 | 18.5 | 1.2 | 11.2 | 9.5 | 1.0 |
| **CXCL5** | 168.3 | 31.1 | 1.1 | 23.4 | 31.2 | 1.0 | 22.4 | 12.6 | 1.0 |
| **CXCL9** | 90.9 | 19.5 | 1.1 | 325.3 | 33.5 | 1.2 | 73.3 | 13.9 | 1.3 |
| **CXCL10** | 190.1 | 118.3 | 5.0 | 104.1 | 52.9 | 1.8 | 27.2 | 18.1 | 1.0 |
| **CXCL11** | 34.1 | 190.1 | 1.3 | 27.0 | 250.7 | 1.3 | 5.0 | 36.9 | 1.1 |
| **CXCL13** | 4.2 | 2.2 | 0.6 | 64.6 | 1.6 | 24.0 | 44.6 | 1.5 | 1.0 |
| **IFNG** | 1.4 | 48.2 | 0.8 | 1.3 | 115.9 | 0.9 | 1.2 | 7.1 | 0.7 |
| **IL1A** | 1.4 | 12.2 | 1.1 | 1.4 | 3.4 | 1.4 | 1.0 | 2.2 | 1.2 |
| **IL1B** | 25.3 | 41.8 | 1.6 | 23.9 | 14.8 | 1.0 | 18.4 | 11.8 | 0.7 |
| **IL1RN** | 40.7 | 66.3 | 8.0 | 15.9 | 30.4 | 2.1 | 9.0 | 15.6 | 0.6 |
| **IL5** | 1.0 | 1.3 | 1.0 | 1.0 | 1.0 | 1.2 | 0.8 | 1.0 | 1.2 |
| **IL6** | 181.5 | 99.5 | 0.9 | 57.7 | 40.8 | 1.0 | 6.4 | 7.0 | 1.0 |
| **IL10** | 1.0 | 1.1 | 1.0 | 1.0 | 1.7 | 1.0 | 1.1 | 2.3 | 1.1 |
| **IL18** | 1.3 | 1.8 | 1.6 | 2.9 | 1.3 | 1.2 | 11.5 | 1.6 | 1.0 |
| **SPP1** | 3.7 | 5.1 | 46.7 | 2.0 | 2.1 | 1.0 | 4.6 | 1.3 | 1.3 |
| **TNF** | 2.1 | 5.4 | 1.6 | 2.2 | 2.6 | 2.2 | 2.2 | 2.1 | 1.3 |
| **Cytokine Receptors and Signaling Molecules** | | | | | | | | | |
|  | **6h** | | | **24h** | | | **48h** | | |
| **Symbol** | **Mus** | **dLN** | **blood** | **Mus** | **dLN** | **blood** | **Mus** | **dLN** | **blood** |
| **CCR1** | 5.4 | 4.0 | 3.3 | 7.8 | 4.6 | 1.4 | 14.4 | 3.9 | 0.7 |
| **CCR2** | 3.0 | 0.7 | 0.7 | 4.1 | 0.8 | 0.2 | 28.1 | 1.7 | 0.5 |
| **CCR5** | 3.8 | 2.0 | 2.5 | 5.8 | 2.8 | 1.3 | 27.8 | 3.3 | 1.3 |
| **CSF1R** | 1.0 | 0.5 | 1.3 | 1.3 | 0.2 | 0.7 | 5.2 | 0.6 | 0.8 |
| **CSF3R** | 2.2 | 2.6 | 1.7 | 2.5 | 2.6 | 1.3 | 3.4 | 2.1 | 0.7 |
| **IFNAR2** | 6.5 | 0.9 | 1.2 | 3.7 | 1.0 | 0.8 | 8.7 | 0.9 | 0.9 |
| **IFNGR1** | 2.1 | 0.9 | 1.3 | 1.6 | 0.7 | 0.5 | 1.6 | 0.9 | 0.5 |
| **IL10RA** | 1.3 | 1.7 | 0.7 | 1.5 | 1.4 | 0.3 | 3.7 | 1.3 | 0.4 |
| **IL10RB** | 2.0 | 0.9 | 1.6 | 1.3 | 0.8 | 0.7 | 3.7 | 1.0 | 0.6 |
| **IL13RA1** | 6.7 | 2.3 | 6.6 | 6.8 | 2.3 | 3.8 | 5.0 | 1.3 | 1.3 |
| **IL15RA** | 2.3 | 3.6 | 2.5 | 3.8 | 3.8 | 2.2 | 1.1 | 1.6 | 1.1 |
| **IL1R2** | 7.0 | 5.7 | 36.7 | 2.6 | 3.2 | 8.3 | 1.5 | 1.7 | 0.8 |
| **IL6RA** | 4.8 | 1.1 | 1.3 | 7.7 | 1.2 | 0.7 | 5.4 | 1.3 | 0.5 |
| **STAT1** | 4.2 | 3.7 | 1.3 | 25.1 | 6.7 | 6.5 | 29.1 | 4.4 | 3.0 |
| **STAT3** | 3.0 | 1.9 | 1.6 | 4.2 | 2.0 | 0.8 | 2.3 | 1.4 | 0.7 |
| **TNFRSF12A** | 1.3 | 2.2 | 1.1 | 4.2 | 1.9 | 0.9 | 0.5 | 1.2 | 1.5 |
| **TNFRSF12A** | 1.4 | 2.2 | 1.2 | 4.4 | 2.0 | 1.4 | 0.6 | 1.4 | 1.2 |
| **TNFRSF1B** | 4.9 | 1.6 | 1.5 | 5.6 | 1.5 | 0.8 | 13.4 | 2.1 | 0.8 |
| **Complement and Antigen Processing/Presentation** | | | | | | | | | |
|  | **6h** | | | **24h** | | | **48h** | | |
| **Symbol** | **Mus** | **dLN** | **blood** | **Mus** | **dLN** | **blood** | **Mus** | **dLN** | **blood** |
| **B2M** | 3.4 | 1.2 | 1.0 | 5.0 | 1.2 | 0.8 | 5.5 | 1.1 | 1.0 |
| **C1QA** | 0.5 | 0.7 | 0.3 | 1.2 | 0.5 | 1.9 | 3.0 | 1.0 | 1.5 |
| **C1QB** | 1.3 | 1.4 | 0.3 | 1.9 | 0.9 | 2.4 | 5.2 | 1.3 | 1.2 |
| **C1QC** | 0.6 | 0.8 | 0.4 | 1.2 | 0.7 | 2.5 | 3.4 | 1.4 | 1.9 |
| **C3** | 5.1 | 1.1 | 7.9 | 8.8 | 1.0 | 3.8 | 6.7 | 0.8 | 2.3 |
| **FCER1G** | 4.8 | 2.3 | 2.3 | 10.9 | 2.8 | 1.3 | 32.4 | 3.8 | 0.8 |
| **FCGR1** | 3.4 | 11.8 | 4.7 | 9.6 | 22.0 | 1.7 | 44.2 | 11.5 | 1.4 |
| **H2-AA** | 1.2 | 0.8 | 0.2 | 1.7 | 0.7 | 0.2 | 2.3 | 0.9 | 0.3 |
| **H2-D1** | 3.7 | 1.1 | 1.1 | 6.7 | 1.4 | 0.7 | 10.4 | 1.4 | 0.8 |
| **H2-EA** | 1.6 | 0.7 | 0.2 | 1.4 | 0.6 | 0.2 | 2.2 | 0.9 | 0.3 |
| **H2-K1** | 5.4 | 1.2 | 0.7 | 8.2 | 1.4 | 0.5 | 11.4 | 1.5 | 0.7 |
| **TAP2** | 5.8 | 1.6 | 0.9 | 10.8 | 2.5 | 0.8 | 4.1 | 1.3 | 0.7 |
| **TAPBP** | 3.3 | 1.9 | 1.0 | 6.4 | 2.6 | 1.1 | 6.1 | 1.7 | 0.7 |
